# Contention over undergraduate medical curriculum content

**DOI:** 10.5116/ijme.5de7.7516

**Published:** 2019-12-16

**Authors:** Stig Andersen, Diana Stentoft, Jeppe Emmersen, Sten Rasmussen, Svend Birkelund, Susanne Nøhr

**Affiliations:** 1 Department of Clinical Medicine, Faculty of Medicine, Aalborg University, Denmark; 2 Department of Health Science and Technology, Faculty of Medicine, Aalborg University, Denmark; 3 Faculty of Medicine, Aalborg University, Denmark

**Keywords:** Contention, undergraduate, medical curriculum content, approaches to teaching and learning, Denmark

## Introduction

Recommendations for medical curriculum content exist in abundance, and each stakeholder has a different view on which curriculum content is more important. Agendas may be tied to education, technology or politics, or they reflect the state-of-the-art of particular medical domains. It is clear that curricula need to adapt to the dynamics of society, changing demographics, health care advancements and the development of new knowledge. To encompass all stakeholder perspectives and to ensure timely prioritisation of knowledge, undergraduate medical curriculum reform should be iterative and theory-led.

Recent reports on teaching palliative medicine in undergraduate medical education provide an example that highlights the importance of continuous undergraduate medical curriculum reform.^1^^,^^2^ A key point is the necessity to continuously assess the degree of detail for core curriculum content and that this assessment is leaning heavily on pedagogy and teaching approaches.^2^

### Medical curriculum content

The rate of development of new knowledge in biomedicine is an ongoing challenge to curricula in medical education and the development of learning objectives. It is clear that the knowledge available in medicine vastly exceeds what an individual can learn.^3^ This raises a key question on how to prioritise the content of the undergraduate curriculum for medical students to become doctors at a basic level balancing all relevant areas within the profession.

The place and content of anatomy in medical education is a classic example.^4^ The needs for and opinions on essential learning in anatomy differ considerably between, e.g., the orthopaedic surgeon and the psychiatrists. Geriatric medicine provides another example.^5^ Frail old patients are frequently admitted to medical care and 41 experts in geriatric medicine representing 29 European countries have set minimum requirements for what they consider adequate training for future doctors in this field. The design was solid using a three-round Delphi method, and it established a European undergraduate curriculum guide in Geriatric Medicine that provided 70 learning outcomes in 10 domains.^5^

The outlined view by the orthopaedic surgeons on learning in anatomy, by the geriatricians on core competencies in medicine for the old, and by the palliative care team on learning needs for end-of-life care sets the scene for undergraduate medical curriculum content contention. In addition, it holds the potential for developing skewed curricula if strategies are not employed to ensure balance and avoid information overload. The Danish author Hans Christian Andersen in the fairy tale “Five Peas from a Pod” puts such suggestions in perspective when he writes; “There were five peas in one pod; the peas were green and the pod was green, and so they believed that the whole world was green - and that was absolutely right!” Hence, the individual recommendations may be flawed by the context, and it is perilous to try to accommodate the recommendations by all specialties and all medical subjects into one undergraduate medical curriculum.

Medical faculty must be aware of the need to counterbalance the tide of subject-specific suggestions for content in curriculum reform. A focus on developing competencies for learning rather than competencies for memorising content may be a lever that can support balancing learning skills against subject-specific curricula content and prepare students at the undergraduate level for lifelong and self-directed learning.^6^^,^^7^ Indeed, the focus of undergraduate medical education has shifted somewhat towards preparing students for postgraduate training rather than on the readiness for independent medical practice.^7^ Hence, there is an ever-increasing need to implement novel models for learning that lean on concepts such as Self-Regulated Learning and maintain a focus on the integration of basic sciences into clinical practice.^8^ Such models should enable students to learn beyond undergraduate training in order to absorb the recommendations by each of the medical specialties that cannot be included in the medical curriculum if this is to be well-balanced and realistic.^9^

We suggest an iterative and theory-led approach to curriculum development. This approach could be illustrated through the curriculum reform at Aalborg University, Denmark, which is the third in nine years. The first call was to design an integrated, spiral curriculum adopting principles of problem-based learning. The second reform was a fine-tuning of design, learning outcomes and assessments strategies to ensure focus on learning rather than memorising facts. The present and third reform is instigated to ensure an explicit pedagogy and learning strategies for improvement of learning skills throughout the curriculum. Our medical curriculum holds a first-year module on learning strategies in order to ensure that students develop competencies for identifying learning gaps, organising learning sessions, and promote reflection on learning outcomes. This design aims to support spiral integration of acquiring competencies for learning across time and disciplines.

### Approaches to teaching and learning

In their argument for increased focus on palliative care in the undergraduate medical curriculum, Brask-Thomsen and colleagues acknowledge the difficulties in assessing the number of lessons.^2^ Yet, they go on to perform a crude count of lessons and conclude based on these numbers that teaching in palliative care does not comply with the recommended number of hours.^2^ This they do without addressing the need for balancing all medical specialties within one curriculum and without considering how teaching and learning may happen in settings which are not scheduled or labelled as a lesson.

Medical schools employ a diversity of methods for teaching and learning with different use of lectures.^3^ For example, learning approaches at Aalborg University aim to promote active learning by including large group and small group case sessions, team-based learning, digital tools, practical skills training, learning in simulated environments, inter-professional learning sessions, clinical placements as well as feedback on portfolios of patient notes taken while engaged in clinical practice. The use of a variety of learning opportunities is an advantage to the students as it allows them to develop their individual learning profiles and in that, they can find learning opportunities to match their needs. However, the use of multiple teaching and learning approaches obviously complicates a direct comparison between medical schools and so to make a more valid comparison would instead require an evaluation of learning outcomes since the majority of learning objectives in curricula are similar across medical schools. This form of comparison would overcome the potential confusion between what is being taught and what the students learn.^3^ Moreover, it would be concerned less with the assumption that number of hours taught equals learning and more with a concern for students’ being prepared to meet challenges of for example palliative care, geriatrics or orthopaedic surgery.

## Conclusions

Undergraduate medical curriculum reform is needed for medical education to adapt to the dynamics of society, health care advancements and the development of new knowledge. The initial claim of this paper was that iterative and theory-led curriculum reforms are needed in undergraduate medical education. Continuous iterations are required to ensure medical curricula, and learning objectives stay abreast with medical advancements and theory-led principles for prioritising knowledge ensures that curricula are not skewed towards one medical specialty over others. In addition, curriculum form must also explicate a commitment to pedagogy. This may guard against disruptive content reform and guide and support students to develop competencies for learning to and prepare them for the postgraduate training and for all the content, which cannot be accommodated in the undergraduate medical curriculum.

### Conflict of Interest

The authors declare that they have no conflict of interest.
